# Neoline from *Aconitum flavum* Hand

**DOI:** 10.1107/S1600536811015170

**Published:** 2011-05-20

**Authors:** Wei Liu, Xiong-Jun Gou, Qin Song, Feng-Zheng Chen

**Affiliations:** a Faculty of Biotechnology Industry, Chengdu University, Chengdu 610016, People’s Republic of China

## Abstract

The title compound, C_24_H_39_NO_6_ [systematic name: (1α,6α,14α,16β)-*N*-ethyl-6,16-dimeth­oxy-4-meth­oxy­methylaconitane-1,8,14-triol], is a C_19_-diterpenoid alkaloid from the roots of *Aconitum flavum* Hand. The mol­ecule has an aconitane carbon skeleton with four six-membered rings and two five-membered rings. Both five-membered rings adopt envelope conformations. Two six-membered rings adopt chair conformations, whereas the other two adopt boat conformations. Intra­molecular O—H⋯O and O—H⋯N and inter­molecular O—H⋯O hydrogen bonds are present in the structure. In the crystal, one methyl group is disordered over two sites with an occupancy ratio of 0.70 (3):0.30 (3).

## Related literature

The title compound is a diterpenoid alkaloid; for the structures of related diterpenoid alkaloids, see: Wang *et al.* (2009 ▶). The title compound had been previously isolated from the roots of *Aconitum carmichaeli* Debx, and the chemical structure was established from NMR and MS data, see: Pelletier & Dailey (1976 ▶). 
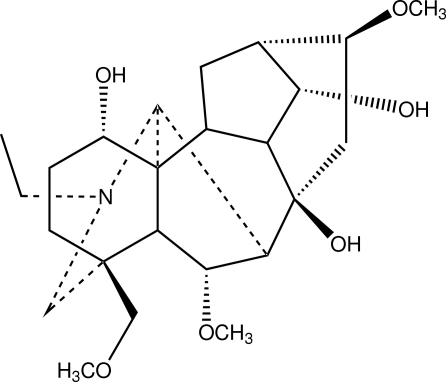


## Experimental

### 

#### Crystal data

C_24_H_39_NO_6_
*M* *_r_* = 437.56
Orthorhombic, 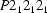

*a* = 9.5423 (6) Å
*b* = 13.4727 (9) Å
*c* = 18.4251 (13) Å
*V* = 2368.7 (3) Å^3^
*Z* = 4
Mo *K*α radiation
μ = 0.09 mm^−1^
*T* = 293 K
0.42 × 0.33 × 0.30 mm

#### Data collection

Oxford Diffraction Xcalibur Eos diffractometer
18153 measured reflections
2422 independent reflections
2130 reflections with *I* > 2σ(*I*)
*R*_int_ = 0.027

#### Refinement

*R*[*F* ^2^ > 2σ(*F* ^2^)] = 0.058
*wR*(*F* ^2^) = 0.177
*S* = 1.09
2422 reflections
295 parameters
H-atom parameters constrained
Δρ_max_ = 1.02 e Å^−3^
Δρ_min_ = −0.18 e Å^−3^

### 

Data collection: *CrysAlis PRO CCD* (Oxford Diffraction, 2009 ▶); cell refinement: *CrysAlis PRO CCD*; data reduction: *CrysAlis PRO RED* (Oxford Diffraction, 2009 ▶); program(s) used to solve structure: *SHELXTL* (Sheldrick, 2008 ▶); program(s) used to refine structure: *SHELXTL*; molecular graphics: *SHELXTL*; software used to prepare material for publication: *SHELXTL*.

## Supplementary Material

Crystal structure: contains datablocks I, global. DOI: 10.1107/S1600536811015170/xu5165sup1.cif

Structure factors: contains datablocks I. DOI: 10.1107/S1600536811015170/xu5165Isup2.hkl

Additional supplementary materials:  crystallographic information; 3D view; checkCIF report

## Figures and Tables

**Table 1:** Hydrogen-bond geometry (Å, °)

*D*—H⋯*A*	*D*—H	H⋯*A*	*D*⋯*A*	*D*—H⋯*A*
O1—H1*A*⋯N	0.93	1.87	2.699 (4)	146
O4—H4*A*⋯O5	0.89	2.33	2.925 (4)	125
O5—H5*A*⋯O1^i^	0.93	1.80	2.682 (4)	158

Symmetry code: (i) 
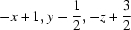
.

**Fig. 1.**
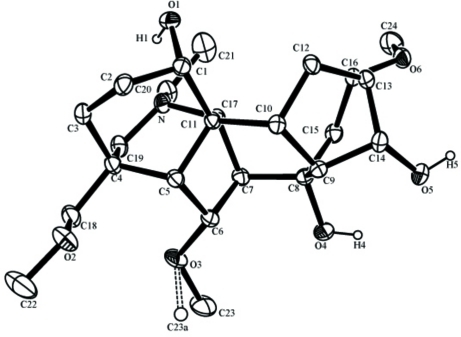
The molecular structure of the title compound with 30% probabiliy displacement ellipsoids for no-H atoms. H atoms have been omitted clarity.

## supplementary crystallographic information

### Comment

The title compound of this report, neoline, is a diterpenoid alkaloid and had been previously isolated from the roots of *Aconitum carmichaeli* Debx. (Pelletier & Dailey, 1976), and its structure was established from the NMR and MS data. The compound itself has analgesic properties, and the plant *Aconitum flavum* Hand has also been therapeutically used to treatrheumatic pain, paralysis due to stroke, rheumatoid arthritis and some other inflammations. In order to obtain further evidence for the exact configuration and conformation of the title compound, we have here determined its singlecrystal structure. The naming and the rings conforming referred to the literature (Wang *et al.* 2009). The molecular structure of the title compound is shown in Fig. 1. Six-membered rings A (C1/C2/C3/C4/C5/C11) and D (C8/C9/C14/C13/C16/C15) adopt boat conformations; six-membered ring B (C7/C8/C9/C10/C11/C17) and six-membered N-containing heterocyclic ring E (C4/C5/C11/C17/N1/C19) adopt chair conformations; five-membered rings C (C9/C10/C12/C13/C14) and F (C5/C6/C7/C17/C11) adopt envelope conformations. The crystal structure contains intermolecular O–H···O and O–H···N hydrogen bonds (Table 1).

### Experimental

The title compound was isolated from the roots of *Aconitum flavum* Hand. And crystals suitable for X-ray structure analysis were obtained by slow evaporation from an acetone solution at room temperature.

### Refinement

Hydroxy H atoms were located in a difference Fourier map and refined as riding in as-found relative positions with *U*_iso_(H) = 1.5*U*_eq_(O). Other H atoms were located geometrically with C—H = 0.96–0.98 Å and refined with a riding model, *U*_iso_(H) = 1.2 *U*_eq_(C). As no significant anomalous scatterings, Friedel pairs were merged.

### Crystal data

**Table:**

C_24_H_39_NO_6_	*F*(000) = 952
*M**_r_* = 437.56	*D*_x_ = 1.227 Mg m^−^^3^
Orthorhombic, *P*2_1_2_1_2_1_	Mo *K*α radiation, λ = 0.71073 Å
Hall symbol: P 2ac 2ab	Cell parameters from 7450 reflections
*a* = 9.5423 (6) Å	θ = 3.0–29.1°
*b* = 13.4727 (9) Å	µ = 0.09 mm^−^^1^
*c* = 18.4251 (13) Å	*T* = 293 K
*V* = 2368.7 (3) Å^3^	Block, colorless
*Z* = 4	0.42 × 0.33 × 0.30 mm

### Data collection

**Table:**

Oxford Diffraction Xcalibur Eos diffractometer	2130 reflections with *I* > 2σ(*I*)
Radiation source: fine-focus sealed tube	*R*_int_ = 0.027
graphite	θ_max_ = 25.2°, θ_min_ = 3.0°
Detector resolution: 10.0 pixels mm^-1^	*h* = −11→11
ω scans	*k* = −15→16
18153 measured reflections	*l* = −17→22
2422 independent reflections	

### Refinement

**Table:**

Refinement on *F*^2^	Primary atom site location: structure-invariant direct methods
Least-squares matrix: full	Secondary atom site location: difference Fourier map
*R*[*F*^2^ > 2σ(*F*^2^)] = 0.058	Hydrogen site location: inferred from neighbouring sites
*wR*(*F*^2^) = 0.177	H-atom parameters constrained
*S* = 1.09	*w* = 1/[σ^2^(*F*_o_^2^) + (0.1203*P*)^2^ + 0.5493*P*] where *P* = (*F*_o_^2^ + 2*F*_c_^2^)/3
2422 reflections	(Δ/σ)_max_ = 0.001
295 parameters	Δρ_max_ = 1.02 e Å^−^^3^
0 restraints	Δρ_min_ = −0.18 e Å^−^^3^

### Special details

**Table:**

Experimental. Because C19-deterpenoid alkaloids from nature have same absolute configurations, although the configuration can?t be determined by present X-ray analysis, it could be confirmed throng comparison with the analogues of the title compound.
Geometry. All e.s.d.'s (except the e.s.d. in the dihedral angle between two l.s. planes) are estimated using the full covariance matrix. The cell e.s.d.'s are taken into account individually in the estimation of e.s.d.'s in distances, angles and torsion angles; correlations between e.s.d.'s in cell parameters are only used when they are defined by crystal symmetry. An approximate (isotropic) treatment of cell e.s.d.'s is used for estimating e.s.d.'s involving l.s. planes.
Refinement. Refinement of *F*^2^ against ALL reflections. The weighted *R*-factor *wR* and goodness of fit *S* are based on *F*^2^, conventional *R*-factors *R* are based on *F*, with *F* set to zero for negative *F*^2^. The threshold expression of *F*^2^ > σ(*F*^2^) is used only for calculating *R*-factors(gt) *etc*. and is not relevant to the choice of reflections for refinement. *R*-factors based on *F*^2^ are statistically about twice as large as those based on *F*, and *R*- factors based on ALL data will be even larger.

### Fractional atomic coordinates and isotropic or equivalent isotropic displacement parameters (Å^2^)

**Table:**

	*x*	*y*	*z*	*U*_iso_*/*U*_eq_	Occ. (<1)
O1	0.7798 (3)	0.62800 (19)	0.74631 (16)	0.0520 (7)	
H1A	0.8642	0.5971	0.7583	0.078*	
O2	1.0936 (4)	0.5268 (3)	0.50259 (19)	0.0754 (10)	
O3	1.0460 (3)	0.3044 (2)	0.6038 (2)	0.0673 (9)	
O4	0.7543 (3)	0.16599 (19)	0.67409 (18)	0.0569 (8)	
H4A	0.6766	0.1394	0.6562	0.085*	
O5	0.4505 (3)	0.1912 (2)	0.68421 (17)	0.0569 (8)	
H5A	0.3618	0.1850	0.7046	0.085*	
O6	0.4605 (3)	0.2734 (2)	0.85463 (15)	0.0546 (8)	
N	0.9676 (3)	0.4789 (2)	0.75691 (18)	0.0444 (8)	
C1	0.7509 (4)	0.5921 (3)	0.6746 (2)	0.0438 (9)	
H1	0.6516	0.6045	0.6647	0.053*	
C2	0.8354 (5)	0.6511 (3)	0.6195 (3)	0.0511 (10)	
H2A	0.8052	0.6340	0.5708	0.061*	
H2B	0.8194	0.7215	0.6267	0.061*	
C3	0.9901 (4)	0.6284 (3)	0.6280 (3)	0.0513 (10)	
H3A	1.0228	0.6564	0.6734	0.062*	
H3B	1.0412	0.6604	0.5890	0.062*	
C4	1.0239 (4)	0.5152 (3)	0.6270 (2)	0.0468 (9)	
C5	0.8907 (4)	0.4534 (3)	0.6083 (2)	0.0401 (8)	
H5	0.8576	0.4682	0.5591	0.048*	
C6	0.9098 (4)	0.3402 (3)	0.6191 (2)	0.0446 (9)	
H6	0.8435	0.3058	0.5873	0.053*	
C7	0.8646 (4)	0.3216 (3)	0.6992 (2)	0.0397 (8)	
H7	0.9377	0.2857	0.7258	0.048*	
C8	0.7251 (4)	0.2647 (3)	0.7017 (2)	0.0411 (8)	
C9	0.6198 (4)	0.3157 (3)	0.6503 (2)	0.0400 (8)	
H9	0.6332	0.2918	0.6005	0.048*	
C10	0.6288 (4)	0.4310 (3)	0.6524 (2)	0.0374 (8)	
H10	0.5943	0.4559	0.6057	0.045*	
C11	0.7761 (4)	0.4789 (3)	0.66603 (19)	0.0353 (8)	
C12	0.5185 (4)	0.4590 (3)	0.7114 (2)	0.0431 (9)	
H12A	0.5619	0.4980	0.7495	0.052*	
H12B	0.4432	0.4976	0.6901	0.052*	
C13	0.4617 (4)	0.3614 (3)	0.7422 (2)	0.0437 (9)	
H13	0.3642	0.3694	0.7578	0.052*	
C14	0.4700 (4)	0.2950 (3)	0.6752 (2)	0.0454 (9)	
H14	0.4044	0.3195	0.6384	0.054*	
C15	0.6700 (4)	0.2528 (3)	0.7800 (2)	0.0460 (9)	
H15A	0.6370	0.1851	0.7856	0.055*	
H15B	0.7483	0.2615	0.8129	0.055*	
C16	0.5518 (4)	0.3230 (3)	0.8043 (2)	0.0409 (8)	
H16	0.5941	0.3800	0.8290	0.049*	
C17	0.8417 (4)	0.4253 (3)	0.7316 (2)	0.0378 (8)	
H17	0.7731	0.4214	0.7711	0.045*	
C18	1.1442 (5)	0.5029 (4)	0.5726 (3)	0.0576 (11)	
H18A	1.2212	0.5466	0.5854	0.069*	
H18B	1.1781	0.4351	0.5733	0.069*	
C19	1.0790 (4)	0.4814 (3)	0.7014 (2)	0.0504 (10)	
H19A	1.1196	0.4157	0.6968	0.060*	
H19B	1.1525	0.5262	0.7171	0.060*	
C20	1.0242 (5)	0.4409 (4)	0.8249 (2)	0.0631 (12)	
H20A	1.1076	0.4785	0.8370	0.076*	
H20B	1.0523	0.3724	0.8177	0.076*	
C21	0.9240 (7)	0.4454 (6)	0.8884 (3)	0.095 (2)	
H21A	0.8893	0.5119	0.8936	0.143*	
H21B	0.9720	0.4261	0.9319	0.143*	
H21C	0.8469	0.4011	0.8799	0.143*	
C22	1.2024 (8)	0.5246 (7)	0.4501 (4)	0.124 (3)	
H22A	1.2768	0.5681	0.4649	0.185*	
H22B	1.1663	0.5462	0.4041	0.185*	
H22C	1.2377	0.4582	0.4457	0.185*	
C23A	1.0543 (11)	0.2077 (7)	0.5829 (9)	0.089 (5)	0.70 (3)
H23A	0.9977	0.1976	0.5405	0.133*	0.70 (3)
H23B	1.0211	0.1658	0.6214	0.133*	0.70 (3)
H23C	1.1500	0.1913	0.5721	0.133*	0.70 (3)
C23B	1.052 (2)	0.254 (2)	0.5398 (13)	0.075 (8)	0.30 (3)
H23D	1.1433	0.2254	0.5342	0.112*	0.30 (3)
H23E	1.0346	0.2995	0.5005	0.112*	0.30 (3)
H23F	0.9824	0.2029	0.5396	0.112*	0.30 (3)
C24	0.5239 (7)	0.2531 (4)	0.9228 (2)	0.0751 (15)	
H24A	0.5958	0.2039	0.9166	0.113*	
H24B	0.4543	0.2287	0.9559	0.113*	
H24C	0.5645	0.3128	0.9418	0.113*	

### Atomic displacement parameters (Å^2^)

**Table:**

	*U*^11^	*U*^22^	*U*^33^	*U*^12^	*U*^13^	*U*^23^
O1	0.0503 (15)	0.0427 (14)	0.0629 (17)	0.0046 (13)	0.0031 (15)	−0.0123 (13)
O2	0.068 (2)	0.094 (3)	0.064 (2)	0.006 (2)	0.0269 (18)	0.0062 (18)
O3	0.0496 (18)	0.0652 (19)	0.087 (2)	0.0161 (16)	0.0240 (17)	−0.0100 (17)
O4	0.0625 (19)	0.0354 (14)	0.0727 (19)	0.0028 (14)	0.0122 (16)	−0.0096 (13)
O5	0.0566 (18)	0.0467 (15)	0.0675 (19)	−0.0189 (14)	0.0077 (15)	−0.0066 (13)
O6	0.0521 (17)	0.0628 (17)	0.0488 (15)	−0.0125 (15)	0.0090 (13)	−0.0020 (13)
N	0.0336 (16)	0.0517 (17)	0.0479 (17)	−0.0041 (15)	−0.0059 (14)	−0.0014 (15)
C1	0.038 (2)	0.0348 (17)	0.058 (2)	0.0039 (16)	0.0029 (18)	−0.0010 (16)
C2	0.048 (2)	0.0353 (19)	0.070 (3)	−0.0028 (18)	0.004 (2)	0.0041 (19)
C3	0.045 (2)	0.045 (2)	0.064 (3)	−0.0098 (18)	0.010 (2)	−0.0011 (19)
C4	0.0333 (18)	0.050 (2)	0.057 (2)	−0.0019 (18)	0.0062 (18)	0.0031 (18)
C5	0.0345 (18)	0.0416 (19)	0.0441 (19)	0.0010 (16)	0.0041 (15)	0.0004 (16)
C6	0.0383 (19)	0.0405 (19)	0.055 (2)	0.0029 (17)	0.0067 (18)	−0.0040 (18)
C7	0.0313 (17)	0.0379 (18)	0.050 (2)	0.0079 (15)	0.0014 (15)	0.0056 (16)
C8	0.0393 (19)	0.0315 (17)	0.052 (2)	0.0017 (16)	0.0057 (17)	−0.0005 (16)
C9	0.0395 (19)	0.0395 (19)	0.0410 (19)	−0.0066 (16)	0.0023 (15)	−0.0056 (16)
C10	0.0327 (18)	0.0351 (17)	0.0443 (19)	0.0006 (15)	−0.0019 (15)	0.0054 (15)
C11	0.0303 (17)	0.0340 (17)	0.0417 (18)	0.0005 (15)	0.0017 (15)	0.0005 (14)
C12	0.0298 (17)	0.0411 (19)	0.059 (2)	0.0032 (16)	0.0020 (17)	0.0038 (17)
C13	0.0317 (17)	0.046 (2)	0.053 (2)	−0.0043 (17)	0.0048 (17)	−0.0044 (17)
C14	0.039 (2)	0.047 (2)	0.050 (2)	−0.0105 (18)	−0.0013 (17)	0.0013 (17)
C15	0.046 (2)	0.0397 (18)	0.052 (2)	0.0004 (18)	−0.0017 (18)	0.0067 (16)
C16	0.0405 (19)	0.0381 (18)	0.044 (2)	−0.0064 (16)	0.0051 (16)	−0.0028 (15)
C17	0.0273 (16)	0.0372 (17)	0.049 (2)	0.0011 (15)	−0.0001 (15)	0.0019 (16)
C18	0.045 (2)	0.060 (3)	0.067 (3)	0.000 (2)	0.017 (2)	0.004 (2)
C19	0.0309 (18)	0.059 (2)	0.062 (2)	−0.0050 (18)	−0.0025 (17)	0.001 (2)
C20	0.052 (2)	0.080 (3)	0.057 (3)	−0.009 (2)	−0.016 (2)	0.008 (2)
C21	0.081 (4)	0.150 (6)	0.056 (3)	−0.024 (4)	−0.017 (3)	0.007 (3)
C22	0.122 (6)	0.154 (7)	0.095 (5)	0.040 (5)	0.066 (5)	0.037 (5)
C23A	0.089 (6)	0.062 (5)	0.115 (11)	0.026 (5)	0.034 (6)	−0.007 (6)
C23B	0.075 (12)	0.088 (17)	0.061 (12)	0.025 (11)	0.027 (9)	−0.008 (11)
C24	0.090 (4)	0.091 (4)	0.044 (2)	−0.003 (4)	0.008 (3)	0.008 (2)

### Geometric parameters (Å, °)

**Table:**

O1—C1	1.434 (5)	C9—H9	0.9800
O1—H1A	0.9332	C10—C12	1.559 (5)
O2—C18	1.414 (6)	C10—C11	1.566 (5)
O2—C22	1.419 (6)	C10—H10	0.9800
O3—C23B	1.359 (17)	C11—C17	1.540 (5)
O3—C23A	1.361 (9)	C12—C13	1.531 (5)
O3—C6	1.415 (5)	C12—H12A	0.9700
O4—C8	1.451 (4)	C12—H12B	0.9700
O4—H4A	0.8861	C13—C16	1.523 (5)
O5—C14	1.421 (5)	C13—C14	1.526 (6)
O5—H5A	0.9305	C13—H13	0.9800
O6—C24	1.421 (6)	C14—H14	0.9800
O6—C16	1.437 (4)	C15—C16	1.539 (5)
N—C20	1.456 (5)	C15—H15A	0.9700
N—C19	1.475 (5)	C15—H15B	0.9700
N—C17	1.478 (4)	C16—H16	0.9800
C1—C2	1.521 (6)	C17—H17	0.9800
C1—C11	1.552 (5)	C18—H18A	0.9700
C1—H1	0.9800	C18—H18B	0.9700
C2—C3	1.516 (6)	C19—H19A	0.9700
C2—H2A	0.9700	C19—H19B	0.9700
C2—H2B	0.9700	C20—C21	1.512 (8)
C3—C4	1.558 (6)	C20—H20A	0.9700
C3—H3A	0.9700	C20—H20B	0.9700
C3—H3B	0.9700	C21—H21A	0.9600
C4—C18	1.534 (5)	C21—H21B	0.9600
C4—C19	1.537 (6)	C21—H21C	0.9600
C4—C5	1.559 (5)	C22—H22A	0.9600
C5—C6	1.549 (5)	C22—H22B	0.9600
C5—C11	1.565 (5)	C22—H22C	0.9600
C5—H5	0.9800	C23A—H23A	0.9600
C6—C7	1.558 (5)	C23A—H23B	0.9600
C6—H6	0.9800	C23A—H23C	0.9600
C7—C17	1.535 (5)	C23B—H23D	0.9600
C7—C8	1.537 (5)	C23B—H23E	0.9600
C7—H7	0.9800	C23B—H23F	0.9600
C8—C9	1.543 (5)	C24—H24A	0.9600
C8—C15	1.543 (5)	C24—H24B	0.9600
C9—C14	1.527 (6)	C24—H24C	0.9600
C9—C10	1.556 (5)		
			
C1—O1—H1A	103.5	C10—C12—H12A	110.3
C18—O2—C22	111.6 (4)	C13—C12—H12B	110.3
C23B—O3—C23A	43.7 (10)	C10—C12—H12B	110.3
C23B—O3—C6	112.4 (9)	H12A—C12—H12B	108.6
C23A—O3—C6	115.9 (5)	C16—C13—C14	112.3 (3)
C8—O4—H4A	109.9	C16—C13—C12	111.8 (3)
C14—O5—H5A	104.7	C14—C13—C12	100.7 (3)
C24—O6—C16	113.7 (4)	C16—C13—H13	110.6
C20—N—C19	109.7 (3)	C14—C13—H13	110.6
C20—N—C17	113.7 (3)	C12—C13—H13	110.6
C19—N—C17	112.2 (3)	O5—C14—C13	118.4 (3)
O1—C1—C2	109.7 (3)	O5—C14—C9	109.7 (3)
O1—C1—C11	113.3 (3)	C13—C14—C9	100.7 (3)
C2—C1—C11	111.4 (3)	O5—C14—H14	109.2
O1—C1—H1	107.4	C13—C14—H14	109.2
C2—C1—H1	107.4	C9—C14—H14	109.2
C11—C1—H1	107.4	C16—C15—C8	117.2 (3)
C3—C2—C1	110.0 (4)	C16—C15—H15A	108.0
C3—C2—H2A	109.7	C8—C15—H15A	108.0
C1—C2—H2A	109.7	C16—C15—H15B	108.0
C3—C2—H2B	109.7	C8—C15—H15B	108.0
C1—C2—H2B	109.7	H15A—C15—H15B	107.2
H2A—C2—H2B	108.2	O6—C16—C13	107.5 (3)
C2—C3—C4	113.5 (3)	O6—C16—C15	110.3 (3)
C2—C3—H3A	108.9	C13—C16—C15	113.8 (3)
C4—C3—H3A	108.9	O6—C16—H16	108.3
C2—C3—H3B	108.9	C13—C16—H16	108.3
C4—C3—H3B	108.9	C15—C16—H16	108.3
H3A—C3—H3B	107.7	N—C17—C7	116.9 (3)
C18—C4—C19	107.2 (3)	N—C17—C11	110.4 (3)
C18—C4—C3	105.5 (3)	C7—C17—C11	100.4 (3)
C19—C4—C3	110.6 (4)	N—C17—H17	109.6
C18—C4—C5	114.0 (3)	C7—C17—H17	109.6
C19—C4—C5	108.5 (3)	C11—C17—H17	109.6
C3—C4—C5	110.9 (3)	O2—C18—C4	108.5 (4)
C6—C5—C4	113.7 (3)	O2—C18—H18A	110.0
C6—C5—C11	102.2 (3)	C4—C18—H18A	110.0
C4—C5—C11	107.6 (3)	O2—C18—H18B	110.0
C6—C5—H5	111.0	C4—C18—H18B	110.0
C4—C5—H5	111.0	H18A—C18—H18B	108.4
C11—C5—H5	111.0	N—C19—C4	112.2 (3)
O3—C6—C5	114.7 (3)	N—C19—H19A	109.2
O3—C6—C7	112.8 (3)	C4—C19—H19A	109.2
C5—C6—C7	104.4 (3)	N—C19—H19B	109.2
O3—C6—H6	108.2	C4—C19—H19B	109.2
C5—C6—H6	108.2	H19A—C19—H19B	107.9
C7—C6—H6	108.2	N—C20—C21	114.6 (4)
C17—C7—C8	108.6 (3)	N—C20—H20A	108.6
C17—C7—C6	105.1 (3)	C21—C20—H20A	108.6
C8—C7—C6	110.4 (3)	N—C20—H20B	108.6
C17—C7—H7	110.9	C21—C20—H20B	108.6
C8—C7—H7	110.9	H20A—C20—H20B	107.6
C6—C7—H7	110.9	C20—C21—H21A	109.5
O4—C8—C7	106.3 (3)	C20—C21—H21B	109.5
O4—C8—C9	108.5 (3)	H21A—C21—H21B	109.5
C7—C8—C9	108.9 (3)	C20—C21—H21C	109.5
O4—C8—C15	107.3 (3)	H21A—C21—H21C	109.5
C7—C8—C15	112.1 (3)	H21B—C21—H21C	109.5
C9—C8—C15	113.5 (3)	O2—C22—H22A	109.5
C14—C9—C8	110.1 (3)	O2—C22—H22B	109.5
C14—C9—C10	103.1 (3)	H22A—C22—H22B	109.5
C8—C9—C10	113.2 (3)	O2—C22—H22C	109.5
C14—C9—H9	110.1	H22A—C22—H22C	109.5
C8—C9—H9	110.1	H22B—C22—H22C	109.5
C10—C9—H9	110.1	O3—C23A—H23A	109.5
C9—C10—C12	102.8 (3)	O3—C23A—H23B	109.5
C9—C10—C11	117.7 (3)	O3—C23A—H23C	109.5
C12—C10—C11	113.2 (3)	O3—C23B—H23D	109.5
C9—C10—H10	107.6	O3—C23B—H23E	109.5
C12—C10—H10	107.6	H23D—C23B—H23E	109.5
C11—C10—H10	107.6	O3—C23B—H23F	109.5
C17—C11—C1	116.4 (3)	H23D—C23B—H23F	109.5
C17—C11—C5	98.4 (3)	H23E—C23B—H23F	109.5
C1—C11—C5	113.1 (3)	O6—C24—H24A	109.5
C17—C11—C10	107.3 (3)	O6—C24—H24B	109.5
C1—C11—C10	106.4 (3)	H24A—C24—H24B	109.5
C5—C11—C10	115.3 (3)	O6—C24—H24C	109.5
C13—C12—C10	106.9 (3)	H24A—C24—H24C	109.5
C13—C12—H12A	110.3	H24B—C24—H24C	109.5

### Hydrogen-bond geometry (Å, °)

**Table:**

*D*—H···*A*	*D*—H	H···*A*	*D*···*A*	*D*—H···*A*
O1—H1A···N	0.93	1.87	2.699 (4)	146
O4—H4A···O5	0.89	2.33	2.925 (4)	125
O5—H5A···O1^i^	0.93	1.80	2.682 (4)	158

Symmetry codes: (i) −*x*+1, *y*−1/2, −*z*+3/2.
